# Gaussian Process Regression Models for Predicting Atomic Energies and Multipole Moments

**DOI:** 10.1021/acs.jctc.2c00731

**Published:** 2023-02-09

**Authors:** Matthew J. Burn, Paul L. A. Popelier

**Affiliations:** Department of Chemistry, The University of Manchester, Oxford Road, Manchester M13 9PL, Britain

## Abstract

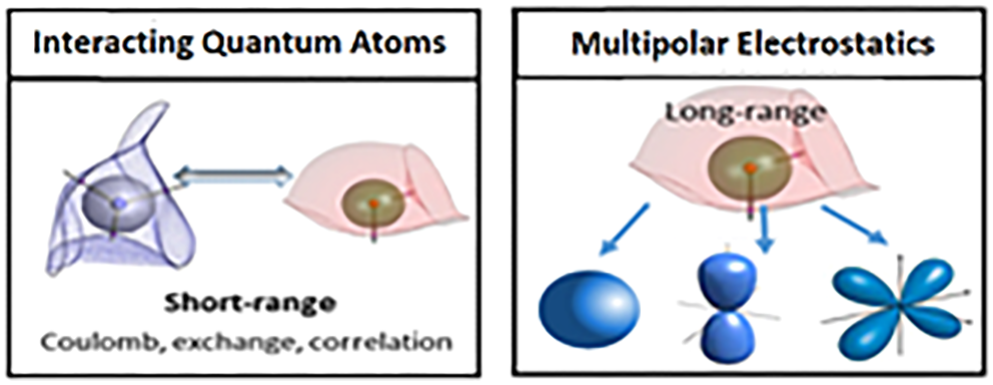

Developing a force field is a difficult task because its design is typically pulled in opposite directions by speed and accuracy. FFLUX breaks this trend by utilizing Gaussian process regression (GPR) to predict, at *ab initio* accuracy, atomic energies and multipole moments as obtained from the quantum theory of atoms in molecules (QTAIM). This work demonstrates that the in-house FFLUX training pipeline can generate successful GPR models for six representative molecules: peptide-capped glycine and alanine, glucose, paracetamol, aspirin, and ibuprofen. The molecules were sufficiently distorted to represent configurations from an AMBER-GAFF2 molecular dynamics run. All internal degrees of freedom were covered corresponding to 93 dimensions in the case of the largest molecule ibuprofen (33 atoms). Benefiting from active learning, the GPR models contain only about 2000 training points and return largely sub-kcal mol^–1^ prediction errors for the validation sets. A proof of concept has been reached for transferring the model produced through active learning on one atomic property to that of the remaining atomic properties. The prediction of electrostatic interaction can be assessed at the intermolecular level, and the vast majority of interactions have a root-mean-square error of less than 0.1 kJ mol^–1^ with a maximum value of ∼1 kJ mol^–1^ for a glycine and paracetamol dimer.

## 1 Introduction

Traditional force fields have long suffered from the limitations inherent to their design. One issue is their fixation with point charges while the accuracy of multipolar electrostatics has been amply documented^1^ for a long while. Another issue is the imprint or impact of strong covalent bonds onto the force field’s image of a molecule and how the latter is modeled. In other words, the Lewis diagram calls the shots in the design of the potential energy expressions of traditional force fields. These expressions cater primarily for the local energy changes associated with strong covalent bonds and create an artificial divide because of bonded and nonbonded interactions in terms of their treatment. An alternative design for a force field is to regard atoms as interacting entities and capture those interactions by the same machine learning (ML) expressions, whether the atoms are bonded or not.

In traditional force fields, more subtle^2^ but still important effects such as an intramolecular hydrogen bond or the interaction between a lone pair and an antibonding π orbital remain absent. However, these interactions can and will be automatically taken into account with ML. This inclusion benefits molecular simulations where such quantum interactions are often essential contributions toward the dynamics of a simulation. As such, ML enables more accurate molecular dynamics (MD) simulations, thereby reducing the need to resort to the alternative *ab initio* molecular dynamics (AIMD), which covers the aforementioned quantum effects but with the drawback of computational cost. While accurate, AIMD simulations cannot be carried out on systems larger than a few dozen atoms at long timescales within a reasonable timeframe. Force fields based on ML take away this limitation.

Biomolecular simulations are typically plagued by the inaccuracies characteristic of classical force fields.^3−7^ Such simulations are reliant on the accurate modeling of intermolecular forces for accurate dynamics.^8,9^ Consequently, inaccurate modeling of these intermolecular forces produces results that are unreliable and often differ, depending on the classical force field used.^9,10^ Many of such inaccuracies stem from the inability of nonpolarizable force fields to effectively model the electrostatic interactions. Hence, extensive research efforts have been made toward producing accurate polarizable force fields.^11−14^

More recently, ML has been successfully employed within force fields^15−23^ including the atomic Spectrum of London and Axilrod-Teller-Muto (aSLATM) potential^24^ and the Faber–Christensen–Huang–Lilienfeld (FCHL) representation.^25^ ML captures quantum data to predict such quantum properties at a fraction of the cost of performing an *ab initio* calculation. Methods such as Gaussian approximation potentials,^26,27^ deep kernel learning,^28^ and neural networks^29,30^ have all shown great promise in accelerating the production of chemically accurate, physically sound properties within a force field framework. The current study focuses on an ML technique known as Gaussian process regression^31,32^ (GPR) (also known as kriging). We believe that the ML method should operate on atomic properties obtained by some (external) partitioning method rather than carry out the atomic partitioning itself. For various reasons,^33,34^ we chose the quantum theory of atoms in molecules^35,36^ (QTAIM) to produce atomic energies and multipole moments^15^ that the GPR then operates on. The use of QTAIM allows for models to be produced on a per-atom basis as properties are also computed on a per-atom basis. This in turn enables the prediction of properties during the simulation at atomic resolution.

The implementation of the GPR models within a force field is undertaken by the in-house software package DL_FFLUX.^37^ This package is based on the DL_POLY^38^ and implements all aspects of the force field FFLUX^22,39^ except its training, which is carried out by the in-house FORTRAN program FEREBUS^40^ and the in-house Python script ICHOR [see the Supporting Information (SI) for details]. The trained GPR models are called within DL_FFLUX such that atomic properties are predicted during the simulation. A GPR model’s input is the nuclear geometry of the given atom’s environment, and its output is a property of that atom. The GPR models thus provide instantaneous, interpolated values for the properties of each atom during a simulation, based on the local environment that the atom experiences. That GPRs can reach high accuracy has also been demonstrated by others.^41,42^ The availability of analytical derivatives yields forces^43^ for the multipolar electrostatics, for the exchange (correlation) interactions and the intra-atomic energies. The former covers polarization effects, not only associated with charge transfer (monopolar), but also with the omnipresent dipolar polarization and even higher ranks, beyond quadrupole moment. The energy decomposition scheme Interacting Quantum Atoms^44^ (IQA) generates all atomic energies because, as a topological method, it is consistent with the QTAIM atomic multipole moments. In other words, all atomic properties come from the same partitioning idea (which is a multidimensional integration in real space rather than in Hilbert space). Thanks to our implementation^45^ in DL_FFLUX of multipolar smooth particle mesh Ewald^46−48^ (SPME) summation, we are able to carry out MD simulations, starting with that of liquid water.^49^ These simulations, which now also extend to molecular clusters and crystalline material, all use nonbonded potential because so far the GPR models are confined to individual molecules only. It is known^50^ that hydrogen-bonded complexes can also be successfully modeled by GPR models, but this accomplishment still needs to be rolled out in the context of simulations. Currently, there is no alternative to GPR models modeling intermolecular repulsion. As a result, the current monomeric modeling invokes an external non-ML potential, such as the Lennard-Jones potential, to take care of nonelectrostatic intermolecular interactions.

The current study demonstrates the use of active learning to produce accurate GPR models for systems of interest to the pharmaceutical industry and ultimately biomolecular simulations. Active learning is a term used to describe a series of algorithms designed to improve the training set of an ML model through iteratively adding unlabeled data points that are expected to increase the predictive accuracy of the model. Active learning has been shown to be a powerful tool in the production of GPR models^51^ for atomistic properties. Its use extends to allow the production of an optimized training set for a single atomic property,^52^ followed by using this given training set to train several GPR models for a variety of atomic properties.

## 2 Methods

### 2.1 (Data) Point Generation

Modeling larger systems naturally requires more (data) points (i.e., molecular geometries) than smaller systems due to the larger sample space. Previous work^51^ has utilized AIMD simulations for the initial point generation. The reason for the use of AIMD is to generate chemically viable geometries at a fixed temperature, free of any potential bias caused by a traditional force field. For small systems, AIMD simulations are a good option as they allow for accurate dynamics and control over the extent of the molecular distortion using temperature. Unfortunately, moving toward larger systems, the AIMD method of point generation does not scale very well and becomes extremely expensive computationally. Coupling the nonlinear scaling of point generation with system size and the need for many more points to describe larger systems necessitates a new approach to point generation.

The most obvious choice is to replace AIMD with classical MD simulations. However, moving to classical MD simulations trades speed for accuracy. In this study, points will be added to the training set based on the geometry alone, which corresponds to unsupervised learning. For this reason, accurate dynamics of the original simulation becomes less of a concern as the simulation is only used to generate geometries and thus the forces (or any other molecular properties) are not required. Furthermore, the energies and forces from the AIMD point generation method are discarded because the “true” wavefunction and atomic properties for each point in the training set are calculated using *ab initio* methods prior to training. In other words, typically the level of theory used by AIMD is lower than that used by the *ab initio* method at the training stage.

The MD software package used in this study is AMBER18^53^ with the general AMBER force field (GAFF2^54^). All systems were run *in vacuo* without periodic boundary conditions and at a fixed temperature. The temperature was set at 300 K for all simulations employing the Langevin thermostat (with ln(γ) = 0.7) and a timestep of 1 fs. Because accurate dynamics were less of a concern than volume of data points generated, no minimization or equilibration step was carried out and the simulations were run for 10,000,000 timesteps (10 ns).

The initial MD trajectory was then split into three sets: training set, validation set, and sample set. This study utilized the so-called per-atom active learning approach where each atom has a unique training set. Alongside this unique training set, each atom also had a unique sample set but all atoms shared the same validation set. To initialize the training set, all input features for all points in the trajectory were calculated. The points corresponding to the minimum feature, maximum feature, and those closest to the mean were added to the training set. This initialization method is known as the min–max–mean initialization and adds up to 3 times *D* points to the initial training set, where *D* is the dimensionality of the system (the number of features). The actual number of initial training points may be smaller than 3*D* because duplicate points are removed if, for example, a single point shares the minimum feature in multiple dimensions. The rationale behind the min–max–mean method is to add points at the boundaries of the search space, and some points in the center, while maintaining linear scaling with respect to the number of features. Alternative approaches to training set initialization, such as the Latin hypercube or minimax distance sampling, may provide better space filling properties but they scale poorly with the number of features and become infeasible for high-dimensional systems. To further assist in filling the search space of the initial training set, 1000 random geometries were added to the initial min–max–mean training set. The sample set and validation set were then selected at random from the remaining points of the initial MD trajectory. In this study 100,000 sample points and 500 validation points were selected for each system.

### 2.2 Feature Calculation

The features used in this study are based upon the atomic local frame^43^ (ALF). Prior to feature calculation, the ALF for each atom of a system must be determined. The ALF defines the axis system for a given atom and consists of the origin atom, an atom defining the *x*-axis, and an atom defining the *xy*-plane. The *z*-axis of the local frame is then defined using the right-hand rule. To define the *x*-axis and the *xy*-plane atoms, the Cahn-Ingold-Prelog rules are used: starting from the origin atom, the atom with highest priority is assigned to the *x*-axis, and the atom with second highest priority is set to the *xy*-plane.

Once the ALF for an atom is defined, the ALF features can be calculated. The first three features are based upon the atoms defining the ALF itself: the first feature is the distance from the origin atom to the atom defining the *x*-axis, the second feature is the distance from the origin atom to the atom defining the *xy*-plane, and the third feature is the angle subtending the vector from the *x*-axis atom to the origin atom and from the origin atom to the *xy*-plane atom. After the first three features there remain 3*N*-9 features, which are the spherical polar coordinates of each non-ALF atom based on the axis system defined by the ALF. The spherical polar coordinates follow the physics convention of (*r*,θ,φ) where *r* is the distance between a given atom, *A*_n_, and the origin atom, *A*_o_, while θ is the polar angle of atom *A*_n_ and φ is the azimuthal angle of atom *A*_n_. Full details of the ALF features are in the SI. It is important to note that the polar angle has a range of [0,π] as it describes the inclination from π/2, whereas the azimuthal angle can range from [−π,π]. The importance of this distinction will become apparent in a later section.

### 2.3 Gaussian Process Regression

GPR is a nonlinear regression machine learning technique. In this study, a single GPR model predicts a single property of a single atom in a system. This means that the number of GPR models associated with a given system is *n*_atom_ × *n*_properties_. A model consists of a training set and a set of hyperparameters, denoted **θ**. The training set is a series of points with a set of inputs, ***X***, and their corresponding outputs, ***y***, where ***X*** is a matrix of size *n*_points_ × *n*_features_ and ***y*** is a vector of length *n*_points_.

A GPR model wholly consists of the training data and the hyperparameters. The latter are used to describe the kernel function (or simply known as kernel) of the GPR model. A GPR interpolates an arbitrary function by fitting the kernel to describe the deviation from the mean. This deviation from the mean is then added to a mean function to provide a prediction,
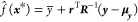
1
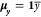
2where *y̅* is the mean of the training outputs, ***y***, and the right term of the sum is responsible for the calculation of the deviation from the mean function for arbitrary point x*. In eq 2, **1** is a column vector of ones. The calculation of the deviation from the mean requires the covariance matrix ***R***, and the covariance vector ***r*** (which is transposed when appearing in eq 1) of the arbitrary point x*. To calculate the covariance matrix and the covariance vector, a kernel is required. The kernel, *k*, is used to calculate the covariance matrix by computing the covariance between the training set (***X***) and itself,
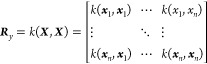
3is used to calculate the covariance matrix and vector as follows:
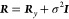
4
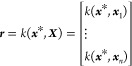
5where the kernel is chosen based on the domain-specific problem, σ^2^ is a noise parameter known as a nugget (which serves the purpose of improving numerical stability), and ***I*** is the identity matrix. The nugget is added to the diagonal of the covariance matrix to improve the numerical stability of inverting the matrix **R** and accounting for noise in the input data. The training data used for model creation are calculated using *ab initio* methods and cleaned prior to training. The input data are cleaned through a process known as scrubbing whereby points with atomic integration errors (*L*(Ω)) greater than 0.001 a.u. are removed from the training set. Therefore, the clean input data will be smooth. As a result, the nugget value can be set as small as 10^–8^. Such a small nugget value is often known as jitter. Previous work has made use of the radial basis function (RBF) kernel, which models the distance between two points in space as a gaussian scaled by the lengthscale parameter ***l***,
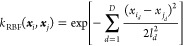
6where *D* denotes the number of dimensions of the system. As stated in Section 2.2, after the first three features, every subsequent group of three features refers to the spherical polar coordinate (with respect to the ALF) of a non-ALF atom. Consequently, every third non-ALF feature is an azimuthal angle φ with a range of [−π,π]. Because the difference between two azimuthal angles can be greater than π radians, the distance between two features may display cyclic properties. In other words, two features may appear further away from each other (larger than π radians) when regarded in a linear rather than cyclic manner (which is the correct view). Thus, modeling this distance with a Gaussian using a simple linear distance measure is inaccurate. The method to fix this issue in previous work has been to modify the RBF kernel to add a cyclic correction to the distance for every azimuthal angle resulting in the RBF-cyclic kernel shown below
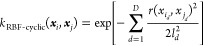
7
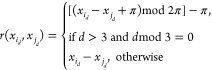
8The current work moves away from the use of the modified RBF kernel used in previous work. The modified RBF kernel is instead replaced with a periodic kernel called the exponential sine-squared kernel,
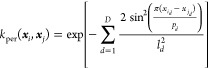
9The exponential sine-squared kernel is similar to the RBF in that a lengthscale parameter, ***l***, is used to scale the difference. However, the kernels differ in the calculation of the difference between the two points: although the RBF kernel computes a linear difference, the periodic kernel replaces this with a sine difference scaled by a period parameter ***p***. Fortunately, we know that the period of the azimuthal angle is 2π. Therefore, this means one parameter fewer to optimize as well as simplifying the kernel computation. Hence, using a well-known trigonometric identity,
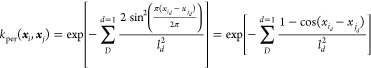
10In summary, the standard RBF kernel (eq 6) is used for all noncyclic dimensions (i.e., the first three features followed by every feature except the azimuthal angle features) while the periodic kernel (eq 10) is used for all cyclic dimensions (i.e., the azimuthal angles). The fact that noncyclic and cyclic dimensions are independent contributions to the kernel, the kernels should be multiplied to provide the total covariance,
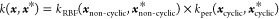
11Because the training set does not change after training, the GPR prediction equation (eq 1) can be simplified by calculating the solution of the matrix multiplication between the inverse covariance matrix and the training outputs vector as follows
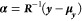
12
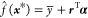
13Simplifying the prediction equation to the form shown in eq 13 improves the scaling of the GPR predictions from *O*(*n*^3^) to *O*(*n*). In other words, the expensive inversion of the ***R*** matrix, which scales as *O*(*n*^3^) is avoided at “run time” when predictions are made. It is a feature of GPR models that the more training points there are in the model, the longer it takes to make a prediction. Therefore, producing the most accurate model with the fewest number of points is highly desirable. This study makes use of active learning to achieve this goal, which will be discussed later in Section 2.4.

Optimization of the kernel hyperparameters is performed by maximizing the concentrated log-likelihood, , which is shown in eq 17. The concentrated log-likelihood is derived from the marginal log-likelihood, , by analytically optimizing the mean. For the sake of simplicity, the value of the constant mean function will be denoted by μ and the analytically optimized concentrated mean value denoted by μ̂,
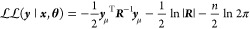
14
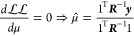
15
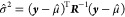
16
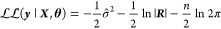
17where *n* is the number of training points and **1** is a vector of length *n*. Maximizing the concentrated log-likelihood to find the optimal hyperparameter vector requires numerical methods. The likelihood function is a highly nonconvex surface with many local optima. To find the global optimum, an evolutionary optimization algorithm called particle swarm optimization^55^ (PSO) is employed. The advantage of a global optimization technique (such as PSO) over local optimizers (such as gradient descent algorithms) is the ability to traverse highly nonconvex surfaces without becoming stuck in local minima.

Inspired by the swarming behavior of birds, PSO consists of a swarm of particles that can communicate with one another to swarm toward the global optimum value. Each particle (*i*) of the swarm has a parameter vector known as the particle’s position, ***p***, and a corresponding velocity, ***v***, which updates the particle’s position every timestep, *t*. To calculate the velocity of particle ***p**_i_* for the next timestep requires three factors: (i) the particle’s current velocity, ***v**_i_*, (ii) the position of the previous personal best (pb) value, ***p***_*i*_pb__, and (iii) the position of the swarm’s previous best global value, ***p***_gb_. These three factors may be summed using three corresponding weights: (i) the inertia weight, ω, (ii) the cognitive learning rate, *c*_1_, and (iii) the social learning rate, *c*_2_. The inertia weight determines the influence of the particle’s current velocity, the cognitive learning rate determines the influence of the pull to the particle’s previously best-known value, and the social learning rate determines the influence of the pull to the swarm’s best-known value. Both the cognitive and social learning rates are combined with a random factor (*r*_1_ and *r*_2_, respectively), where *r*_*k*_ ∈ [0,1], to prevent stagnation,
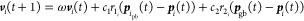
18The GPR and PSO algorithms are implemented by the in-house GPR engine software package FEREBUS. Full details of the settings of all PSO parameters are in the SI.

### 2.4 Active Learning

Active learning^56−59^ is the process of selecting unlabeled data points (i.e., without output value) from a sample set that will improve the training set in the subsequent iteration. Active learning reduces the number of *ab initio* calculations required, as well as producing minimal training sets. A minimal training set is a training set that achieves a certain accuracy in the smallest number of points. This is especially important in the context of GPR models because the prediction time increases with the size of the training set as well as the model’s physical size and thus hardware memory requirements.

Many active learning methods have been developed to solve domain-specific problems. Some active learning methods can become expensive to run when moving toward larger training and sample sets. Previous work focused on the maximum expected prediction error^60^ (MEPE) active learning method, which requires the computation of both the expected cross-validation error and the predictive variance of each sample point. The sample set in this study is orders of magnitude larger than that used in our previous work. Therefore, the active learning method has been simplified to the highest variance method. The highest variance^61^ active learning method calculates the predictive variance of each point in the sample pool selecting the points with the highest variance to add to the training set,
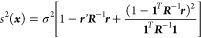
19where σ^2^ is the model variance, **1** is a column vector of ones with length *n*_train_, and the *T* again marks the transpose. The further away a sample point is from the training set, the higher the predictive variance will be and therefore the more likely it will be chosen by the active learning method, that is, included in the training set. The highest variance method favors exploration over exploitation, which could be seen as a disadvantage. Continually favoring exploration would result in a training set where the training points are far apart from one another. Therefore, parts of the potential energy surface where fine detail may be required (such as minima and transition states) may not be sampled as highly as if an exploitative method was used. However, in our case, exploring geometries that are currently not close to the training set, and adding them to the training set, guarantees successful MD runs. Indeed, an MD run can demand a prediction of the GPR model for a spurious geometry, that is, one far away from the original training set.

The mechanism of a failing MD run can be explained as follows. A characteristic of using a constant value as a mean function for the GPR model, is that predictions for points far away from the training set become this constant value. Thus, the gradient associated with this value is zero; if the value is an intramolecular energy then the gradient becomes a force applied to a nucleus due to a change in intramolecular energy. It is undesirable for such a force to vanish during an MD simulation because the absence of these forces means that atoms are not held together anymore as a molecule. Consequently, a force caused by intermolecular (e.g., electrostatic) interaction may pull an affected atom in a given molecule toward another molecule. This effect is devastating for the MD simulation. Adding points further out from geometries, typically seen within normal MD simulations, prevents such situations from occurring.

The atomic property prediction for each atom in a system is independent. Therefore, the training set for each atom in a system and, in fact, each atomic property can be unique. Furthermore, as each training set is unique, each atom’s active learning run is also unique. This allows for the optimization of the training set on a per-atom basis, which we coin per-atom active learning.

All active learning and *ab initio* interfaces are implemented in the in-house pipeline software ICHOR, which allows for highly parallel execution on HPC clusters. A flowchart of ICHOR is shown in the SI.

### 2.5 Atomic Properties

The output properties for the GPR models are atomic properties calculated using *ab initio* methods. In our previous work using active learning, the atomic property being modeled was the IQA energy. The IQA energy is used in atomic simulations for intramolecular interaction, that is, taking care of the internal energy balance of a flexible molecule. The IQA energy captures the intra-atomic energy along with the interaction energy with all atoms other than a given atom *A* in the molecule,
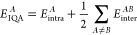
20
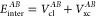
21where *E*_intra_^*A*^ is the intra-atomic energy of atom *A*, *V*_cl_^AB^ is the classical potential energy between atoms *A* and *B*, and *V*_xc_^A^ is the exchange-correlation potential energy between atoms *A* and *B*. Explicit formulae defining these energies can be found in the original IQA publication^44^ and in work^62^ that made IQA compatible with DFT, for the first time. In an MD simulation, the forces to apply to each atom can be calculated from the derivative of the predicted energy allowing the modeling of a single molecule *in vacuo*.

FFLUX is a polarizable force field, which requires electrostatic information to cover intermolecular interactions. Short-range electrostatics are captured by the IQA energy, specifically in the interaction energy (*E*_inter_^*AB*^) of the IQA energy. For long-range electrostatic interactions, FFLUX uses multipole moment predictions^63−65^ from GPR models as an input^45^ to the SPME method.^48^ This allows FFLUX to capture monopole–monopole, monopole–dipole, dipole–dipole, etc. interactions using multipole moments up to hexadecapole moments (*L*′ = 4).

Training multipole moment models is similar to training IQA energies. In fact, the training process is identical, other than for the replacement of the training outputs by the specific multipole moment being trained. It is important to note that it is required that the multipole moments are in the ALF because the features are expressed with respect to the ALF. The multipole moments are typically computed in the global frame so a rotation to the ALF is needed prior to training. Then, when using the GPR multipole moment models in a simulation, the multipole moments must be expressed with respect to the global frame. Thus, the multipole moments must be rotated from the predicted local frame moments back to the global frame.

Quantum chemical calculations were carried out using the program^66^ GAUSSIAN09 at B3LYP/6-31+G(d,p) level of theory, while the QTAIM calculations were carried out using the external program^67^ AIMAll19.

Numerical errors in the computation of the atomic properties cause an integration error to the training input. Certain geometries may result in an especially large integration error and may not be suitable for use in the GPR model. Such geometries must thus be removed from the training set prior to training, in a process known as scrubbing. Points are scrubbed from the training set if the point’s integration error is greater than the threshold of 0.001.

## 3 Results and Discussion

### 3.1 Predictive Accuracy

The first step in validating a model is to test the predictive accuracy of each model against a validation set. Full details of the distortions used to generate each model and the number of training points are in the SI. The validation set is selected at random from the initial AMBER trajectory, after removing the points assigned to the training and sample sets. For this study, a validation set size of 500 points was chosen, and the atomic properties for all 500 points were calculated. We first define the individual prediction error (PE) for a single model, that is, for a given atom and given property. This prediction error is then calculated, for each point in the validation set, as the difference between the true value of the validation point, *f*(***x***), and the predicted value of the validation point, *f̂*(***x***),
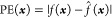
22

**Figure 1:**
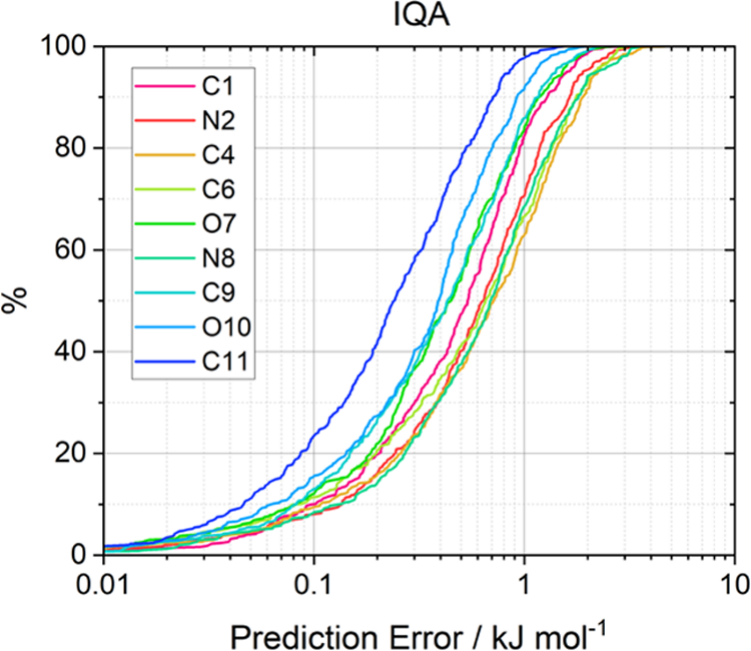
*S*-curves showing the individual atom IQA prediction error for a glycine AMBER 300 K model. Hydrogens are not shown here for clarity but can be found in Figure S4.1 in the SI.

**Figure 2:**
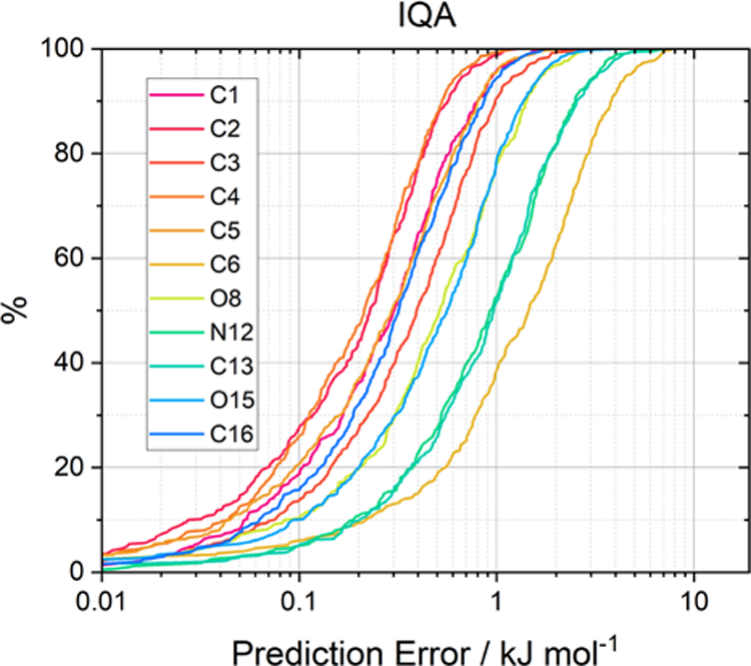
*S*-curves showing the individual atom IQA prediction error for a paracetamol AMBER 300 K model. Hydrogens are not shown here for clarity but can be found in Figure S4.2 in the SI.

The total prediction error of a given property, for each system, then involves a sum over atoms. In particular, we take the difference between the sum of the true values and the sum over the predicted values,
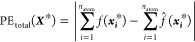
23where X* is the collection of feature vectors for each atom within a given molecule and ***x***_i_^*^ is the feature vector of atom *i* within this molecule. The total prediction error (PE_total_, eq 23) is a useful metric for evaluating the overall accuracy of a model while individual prediction errors (PE, eq 22) allow for the identification of particularly poorly performing models. Plotting the prediction errors against the percentile of the validation point results in an *S*-curve. The *S*-curve is a useful visualization of both the average error (although not explicitly shown) and the distribution of errors across a validation set. Figures 1 and 2 show the *S*-curves for the IQA prediction errors of the glycine and paracetamol models, respectively. The *S*-curves for the remaining systems (aspirin, alanine, glucose, and ibuprofen) are in the SI.

All six systems demonstrate excellent predictive accuracy, with the overwhelming majority of atoms displaying prediction errors below 1 kJ mol^–1^ for 50% of the validation set. Almost all models are below 1 kcal mol^–1^ for 90% of the validation set except for 6 atoms (C3, C8, C12, H23, H27, and C28; atom numberings can be found in the SI) in the ibuprofen model, the worst of which reaches the 1 kcal mol^–1^ threshold at 48% of the validation set.

**Figure 3:**
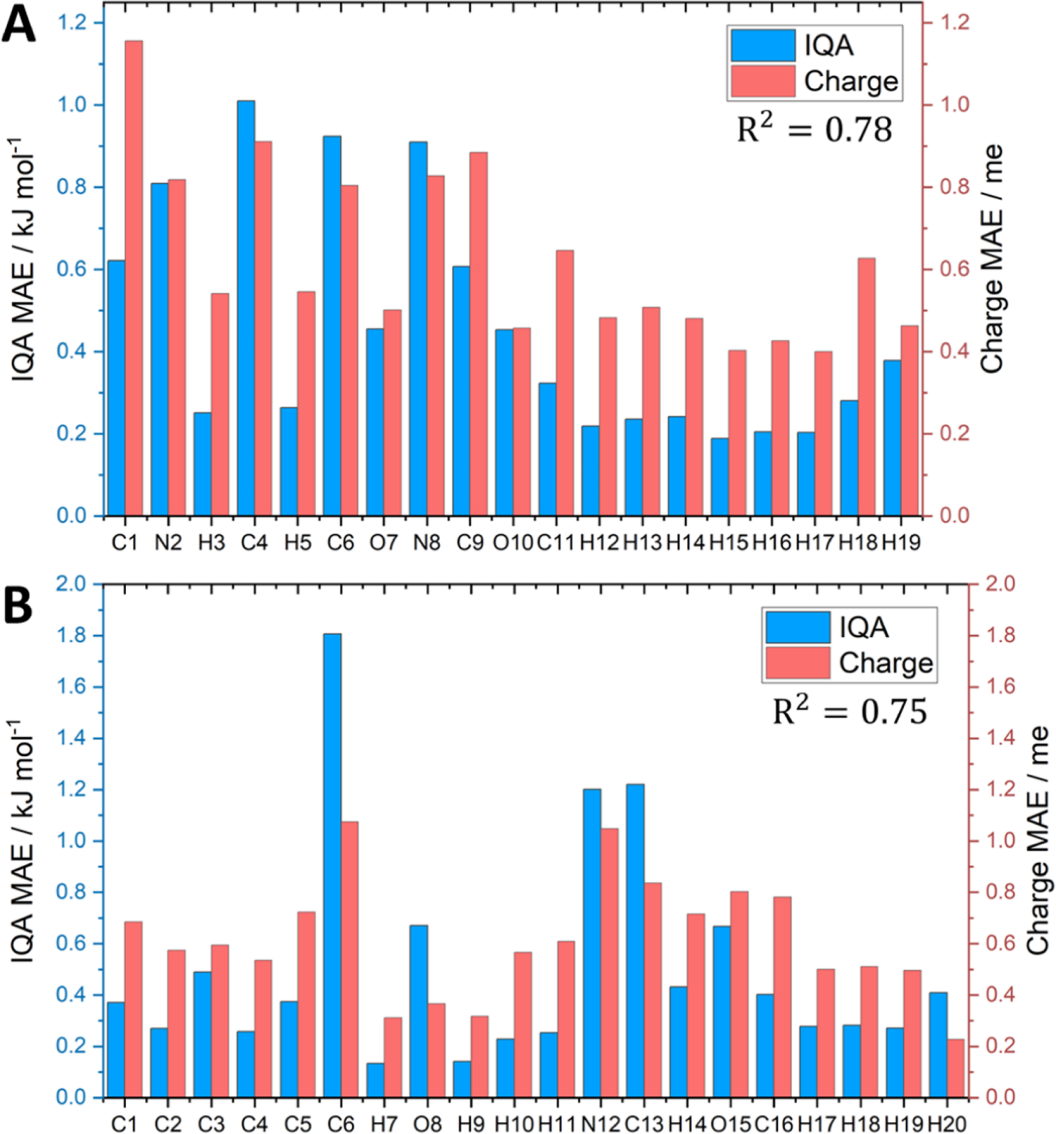
Comparison between IQA and charge predictions for (A) glycine and (B) paracetamol. Both the IQA (kJ mol^–1^) and charge (milli-electron, me) training sets share the same geometries because training set input geometries taken from an active learning run, exclusively using IQA models, are then transferred to produce GPR models for atomic charge.

It is not immediately clear from IQA *S*-curves alone how well a training set, produced by active learning on IQA models, will transfer to multipole moment models. The predictive accuracy of both models must be compared to determine the validity of utilizing a model, created through active learning of the IQA energy, for the multipole moments. Figure 3 shows such a comparison between the mean absolute errors (MAE) of the IQA models (used to create the *S*-curves above) and the charge models (using the IQA training inputs) for the glycine (Figure S7a) and paracetamol (Figure S7b) models.

For both glycine and paracetamol, Figure 3 demonstrates that there is a broad positive correlation (Pearson correlation coefficients *r*^2^ > 0.75) between the predictive accuracy of the IQA models and the charge models. Because IQA prediction errors provide a good insight into the corresponding charge prediction it is unnecessary to produce multipolar models for each iteration of an active learning run. Instead, it is acceptable to infer the multipolar prediction errors from the IQA prediction errors. This insight removes the need to perform a unique active learning run for each atomic property. Rather, active learning should be performed on IQA energy models only, then using the final training set, produce models for the multipole moments once a satisfactory IQA prediction error is reached.

**Figure 4:**
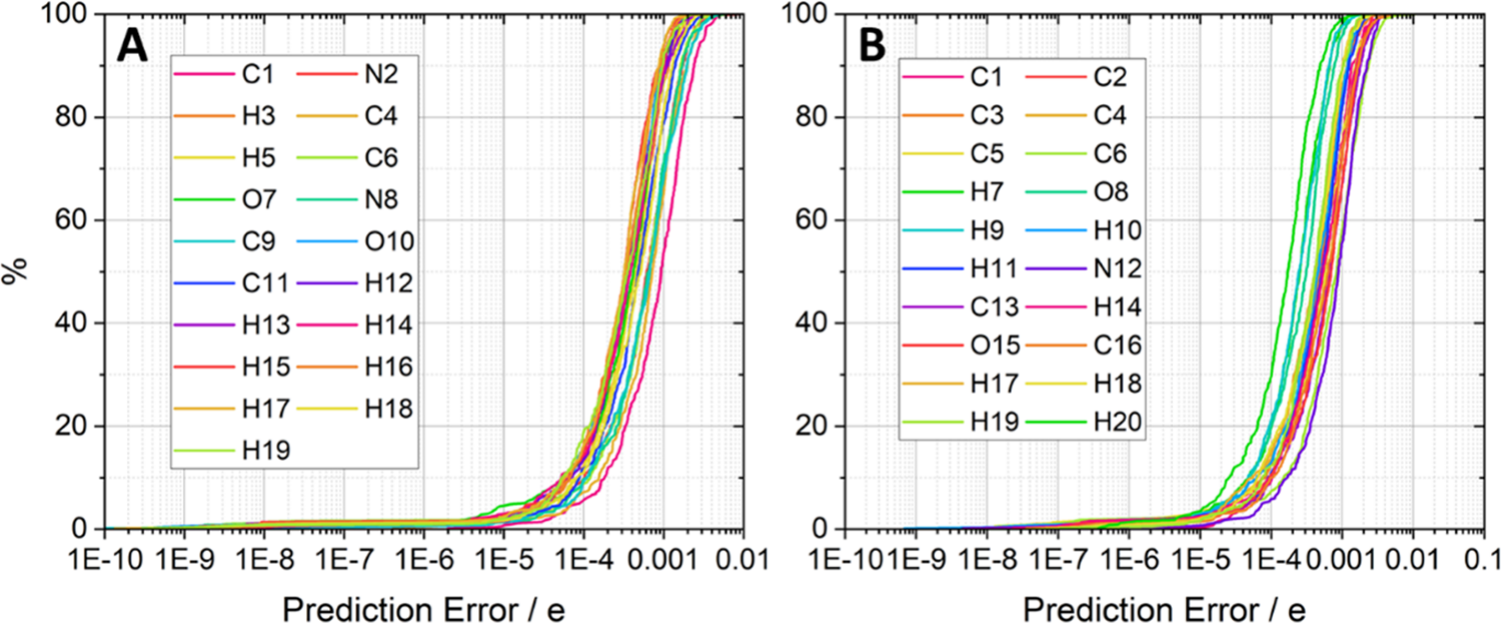
*S*-curves for the (A) glycine and (B) paracetamol atomic charge prediction errors.

Once produced, the multipole moments may undergo the same prediction error analysis as the IQA energies. Unlike IQA energies, multipole moments (excluding charge) are not scalar quantities but are predicted as directional quantities. Therefore, for reasons that are discussed in detail in the next section, *S*-curve analysis provides less insight into their predictive accuracy. However, the charge multipole moment is a scalar quantity with an insightful *S*-curve analysis (Figure 4).

For completeness, the remaining multipole moment *S*-curves for the glycine and paracetamol models shown in Figure S8 are in the SI.

### 3.2 Electrostatic Interactions

For scalar quantities such as energy and charge, computing the raw prediction errors is generally sufficient because the lower the prediction error, the better the model predicts the atomic property. However, for higher-rank multipole moments, this is not necessarily the case because the absolute value of each multipole moment is only half of the picture, the other half being the orientation of the multipole moment. Both magnitude and orientation contribute to the predictive performance of the multipole moment contributions toward the electrostatic interaction. To test the accuracy of these multipole moments, a dimeric validation set is constructed and the multipole moment for each atom in the monomeric state is computed. The dimer configurations were created manually through reflection, rotation, and translation, which enabled the selection of configurations that avoid short-range convergence issues. We quantify the performance of each multipole moment model by comparing the interaction energies produced when predicting multipole moments of dimers in DL_FFLUX. In particular, we compare the interaction energy calculated from the true moments of each atom in one monomer and the true moments of each atom in the other monomer with the same interaction energy but then calculated from the predicted moments. It should be understood that we are comparing the electrostatic energy at the level of the multipolar energy only rather than the full electrostatic energy because we ignore the penetration energy that typically arises from the superposition of monomeric wavefunctions.

The interactions of the multipole moments between each molecule may be controlled using the *L*′ value. An *L*′ = 0 value signifies that only point charges are interacting and we are investigating up to hexadecapole–hexadecapole interactions (*L*′ = 4). Note that L′ = 4 actually includes all multipole–multipole interactions up to (and including) hexadecapole moment. A good measure for the accuracy of a model in predicting the interaction energy is the root-mean-square error (RMSE),
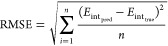
24where *E*_int_pred__ is the predicted interaction energy for a given atom pair, *E*_int_true__ is the true interaction energy between these same atoms, and *n* is the number of validation points used for the predictions.

**Figure 5:**
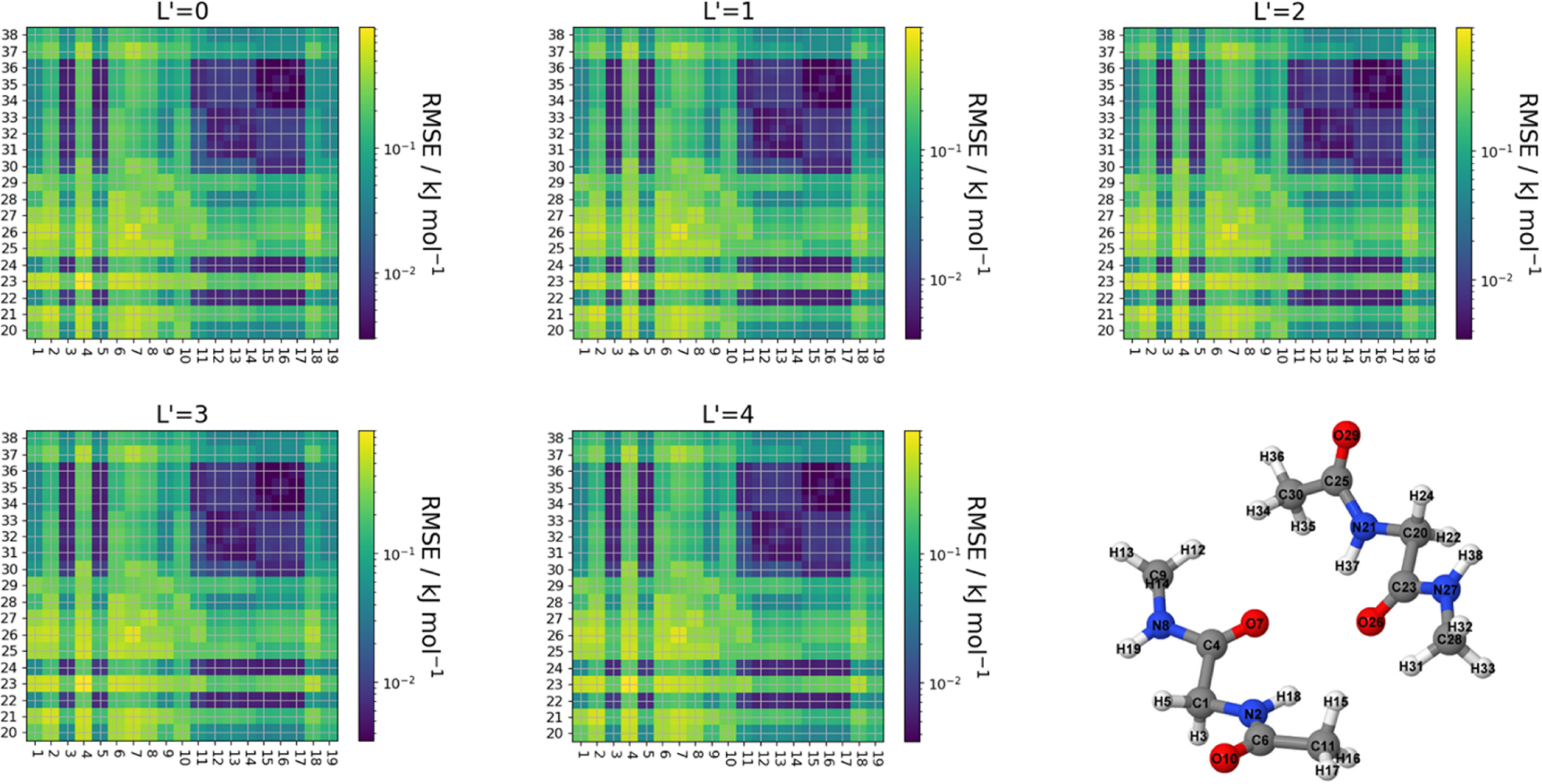
Heatmap showing the RMSE of the interaction energy between each pair of atoms occurring in the glycine dimer for increasing values of *L*′ alongside the atom labeling used for the dimer configuration. Blue indicates a lower RMSE value, while yellow indicates a higher RMSE value.

**Figure 6:**
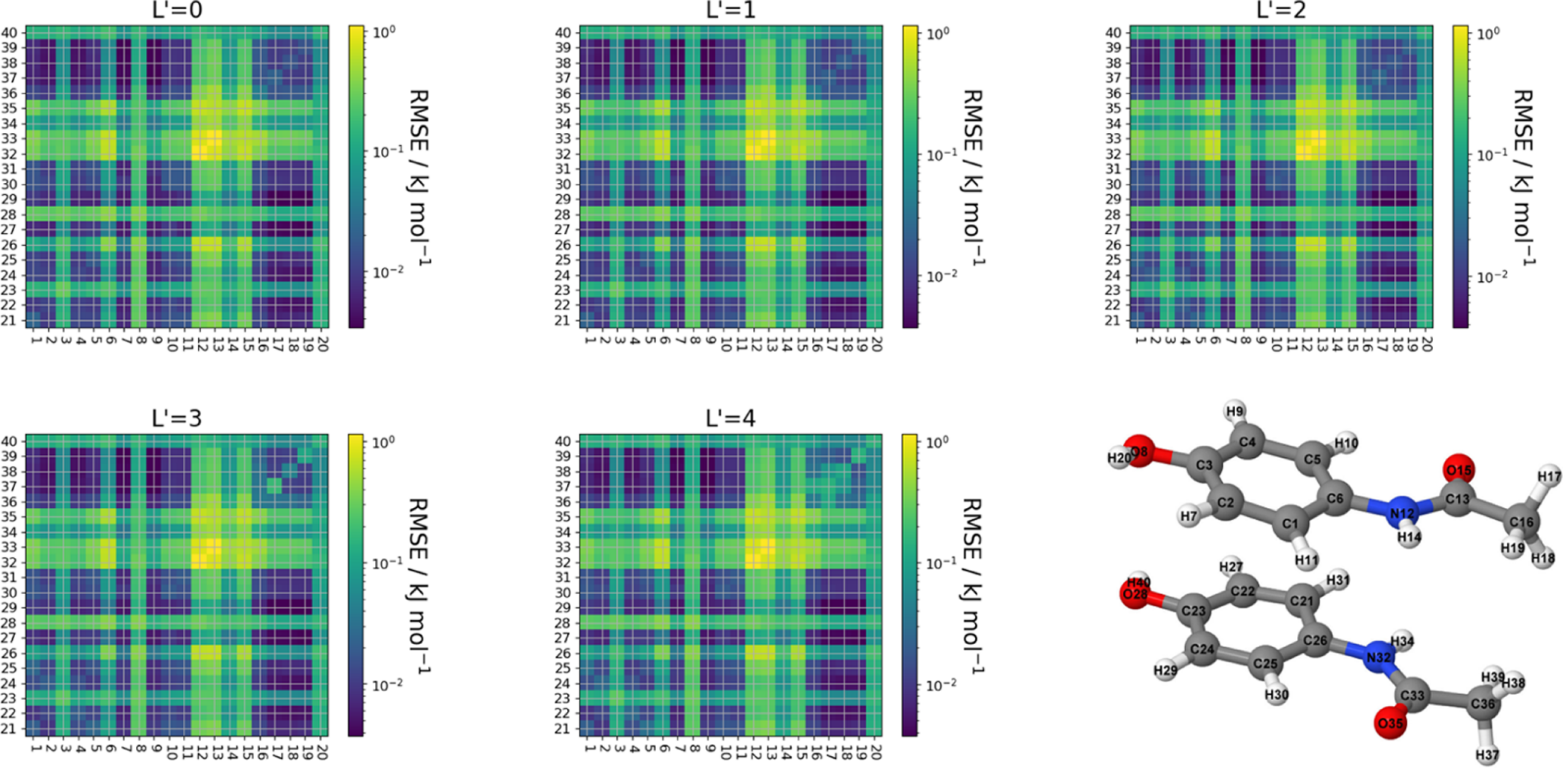
Heatmap showing the RMSE of the interaction energy between each pair of atoms occurring in the paracetamol dimer for increasing values of *L*′ alongside the atom labeling used for the dimer configuration. Blue indicates a lower RMSE value, and yellow indicates a higher RMSE value.

By comparing the interaction energies between each atom of a dimer, we can produce a heatmap showing which interactions are best predicted based on the RMSE values for each atom–atom interaction. Figure 5 shows the (logarithmic) interaction heatmaps of glycine, and Figure 6 shows those of paracetamol showing the RMSE value for every interaction between each atom of the dimer configuration.

As can be seen from Figures 5 and 6, the vast majority of interactions have an RMSE of less than 0.1 kJ mol^–1^ with a maximum RMSE of roughly 1.1 kJ mol^–1^. It is worth noting that interactions such as C4–C23 in glycine and C12–C33 in paracetamol (both having an RMSE greater than 1 kJ mol^–1^) have absolute interaction energies around 500 kJ mol^–1^. Therefore, an error of 1 kJ mol^–1^ corresponds to a ∼0.2% error. Second, we note that the heatmaps change very little between the *L*′ values for a given molecule, with the exception of the two methyl interacting in the paracetamol dimer.

In summary and in general, the two main drawbacks of our methodology both have to do with computational demand. The calculation of the atomic properties is expensive. Second, the CPU time it takes to perform the active learning is amplified by the previous demand because we need to calculate new IQA energies and then retrain at each learning iteration. However, the latter drawback is mitigated somewhat by taking advantage of multiple cores and adding multiple points per iteration.

## 4 Conclusions

The force field FFLUX uses Gaussian process regression (GPR) to learn the energies and multipole moments of quantum topological atoms. These atoms are space filling and together make up six molecules (peptide-capped glycine and alanine, glucose, paracetamol, aspirin, and ibuprofen) that we have successfully trained for using the in-house program FEREBUS and its associated pipeline ICHOR. We distorted these molecules to a fair degree, including full methyl rotations, with an eye on covering many configurations from an AMBER-GAFF2 molecular dynamics run. As such, FFLUX has now been developed beyond its original action radius of normal-mode-distorted minimum energy geometries. The topological partitioning method offers the advantage of consistent atomic energies and multipole moments, both from the same volume integration over atomic volumes. Moreover, GPR needs fewer training points than neural networks, and when combined with active learning, manages to introduce no more than about two thousand training geometries. This methodology can handle systems of dimension of about 50 D (glycine), returning almost 90% of the validation set geometries with an energy prediction error of less than 1 kcal mol^–1^. As the dimensionality of the systems increases, this performance deteriorates, with the exception of glucose (66 D), to just under 20% for the 93-dimensional system ibuprofen. The prediction of electrostatic interaction, at the level of multipolar electrostatics only, can be assessed at the intermolecular level and the vast majority of interactions have a root-mean-square error of less than 0.1 kJ mol^–1^ with a maximum value of ∼1.1 kJ mol^–1^ for a glycine and paracetamol dimer. Finally, a proof of concept has been reached for utilizing a model produced through active learning of one atomic property to produce models for all atomic properties by showing the existence of a broad positive correlation between the predictive accuracy of the atomic energy models and the charge models. As a result, active learning should be performed on energy models only, and the resulting model utilized to produce multipolar models for the final training set once the energy prediction errors are satisfactory.

## Supporting Information Available

Labeled geometries, model sizes, mist plots, *S*-curves, atomic local frame features, particle Swarm optimization, and ICHOR (PDF)

## Notes

The data that support the findings of this study are available from the corresponding author upon reasonable request.
