# Supplementary material for: Gaussian Process Regression Models for Predicting Atomic Energies and Multipole Moments
Source: J Chem Theory Comput. 2023 Feb 9;19(4):1370–80. doi: 10.1021/acs.jctc.2c00731 (PMC9979601; doi:10.1021/acs.jctc.2c00731)
Supplement: Supplementary file 1 — ct2c00731_si_001.pdf [file ct2c00731_si_001.pdf]

Supporting Information

Gaussian Process Regression Models for Predicting Atomic Energies and Multipole Moments

Matthew Burn and Paul Popelier *

Department of Chemistry, The University of Manchester, Manchester, M13 9PL, Britain

*To whom correspondence should be addressed:
Phone: +44 161 3064511. E-mail: pla@manchester.ac.uk

Table of Contents

1	LABELLED GEOMETRIES	2
2	MODEL SIZES	5
3	MIST PLOTS	7
4	S-CURVES	10
5	ATOMIC LOCAL FRAME FEATURES	15
6	PARTICLE SWARM OPTIMISATION	16
7	ICHOR	17

1 Labelled Geometries

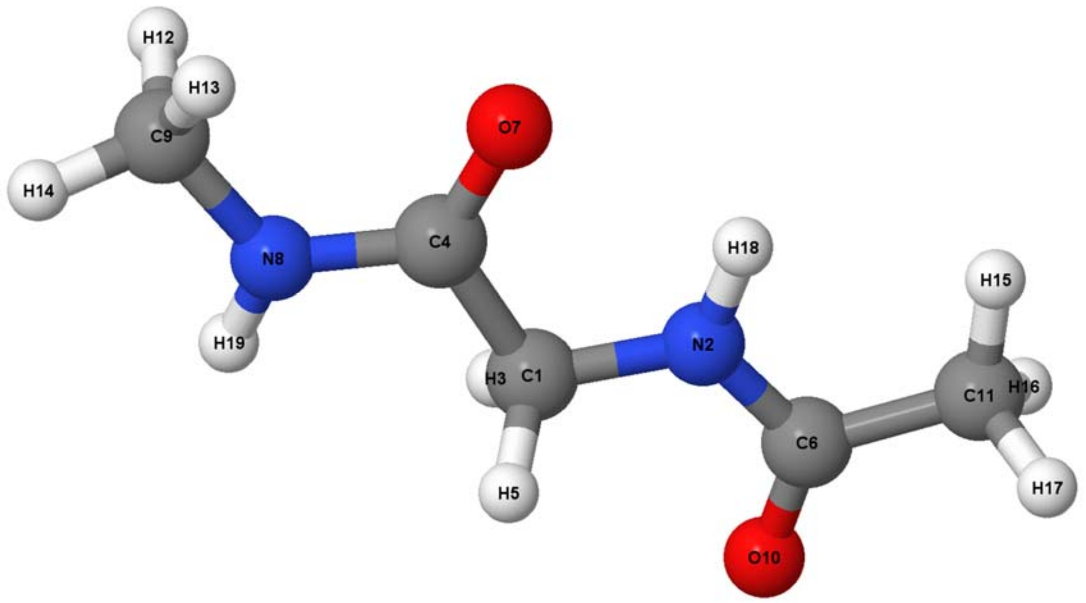

Figure S1.1 Atom labelling of the glycine models.

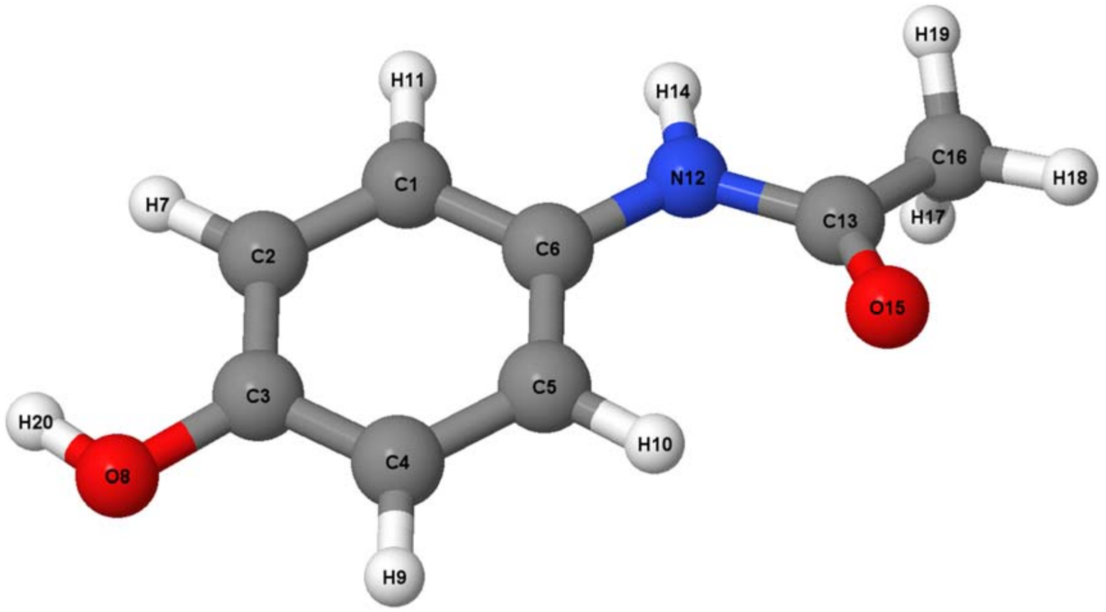

Figure S1.2 Atom labelling of the paracetamol models.

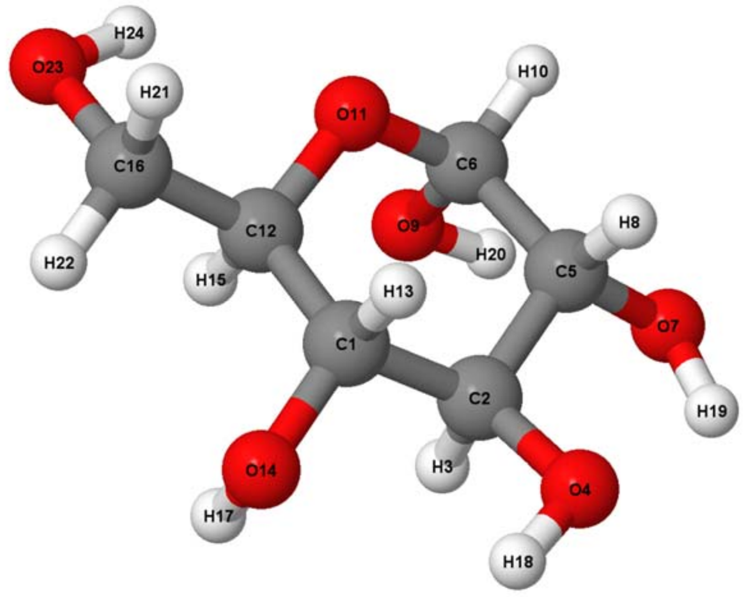

Figure S1.3 Atom labelling of the glucose models.

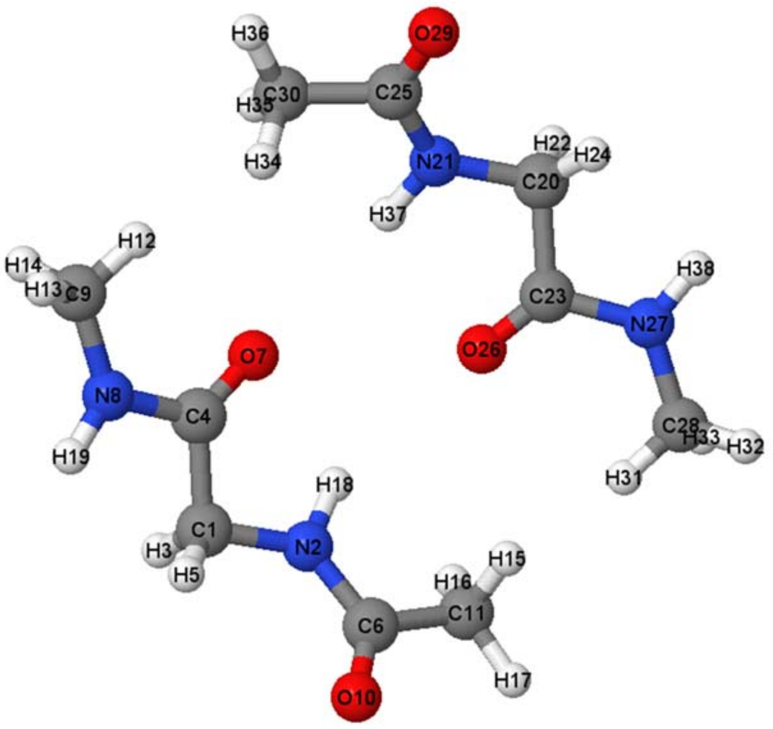

Figure S1.4 Atom labelling of the glycine dimer.

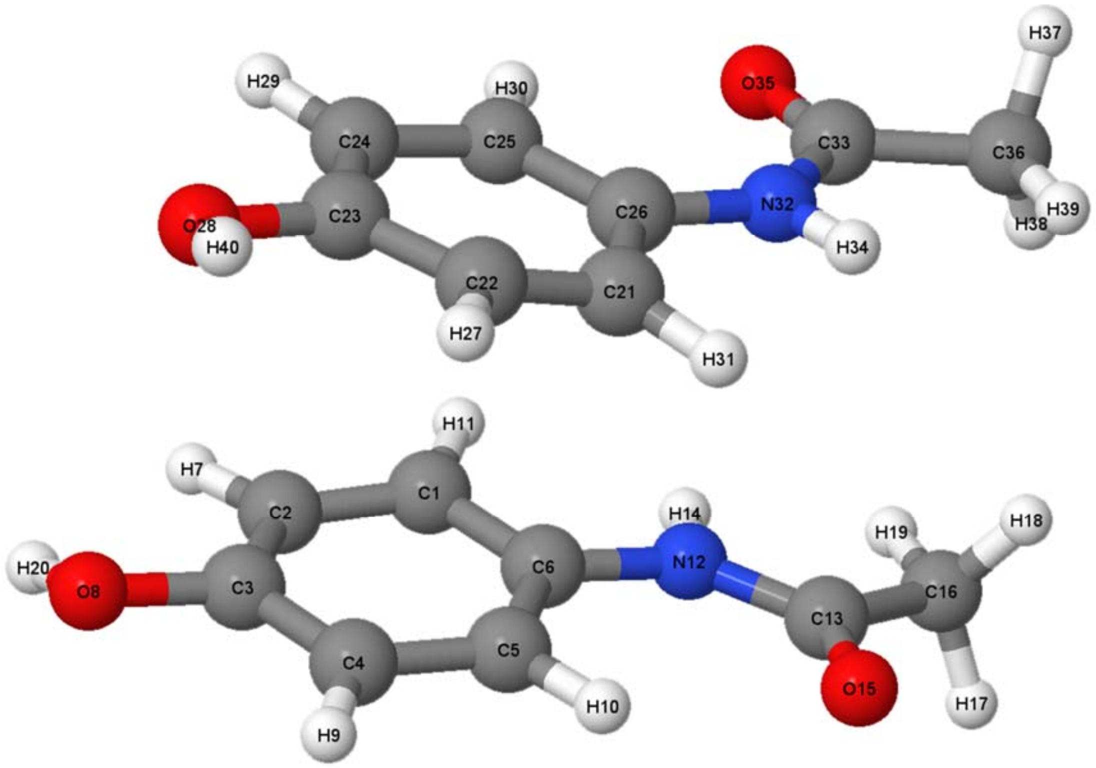

Figure S1.5 Atom labelling of the paracetamol dimer.

2 Model Sizes

Table S1. Number of training points for each atom in the glycine model (19 atoms, 51 features).

Atom Name	Number of Training Points
C1	2130
N2	2144
H3	2133
C4	2133
H5	2134
C6	2139
O7	2130
N8	2127
C9	2120
O10	2136
C11	2126
H12	2117
H13	2118
H14	2128
H15	2136
H16	2123
H17	2127
H18	2127
H19	2127

Table S2. Number of training points for each atom in the paracetamol model (20 atoms, 54 features).

Atom Name	Number of Training Points
C1	2148
C2	2142
C3	2143
C4	2145
C5	2154
C6	2143
H7	2139
O8	2141
H9	2137
H10	2140

H11	2143
N12	2137
C13	2130
H14	2129
O15	2129
C16	2129
H17	2126
H18	2128
H19	2128
H20	2125

Table S3. Number of training points for each atom in the glucose model (24 atoms, 66 features).

Atom Name	Number of Training Points
C1	2163
C2	2182
H3	2167
O4	2182
C5	2149
C6	2168
O7	1630
H8	2167
O9	2158
H10	2173
O11	2172
C12	2159
H13	2173
O14	2154
H15	2716
C16	2178
H17	2118
H18	2150
H19	2132
H20	2113
H21	2170
H22	2155
O23	2135
H24	2145

3 Mist Plots

Mist plots are a convenient way to visualise the distortion of a molecule. The figures below show examples of ball-and-stick representations combined with a distribution ("mist") consisting of every point (i.e. molecular geometry) in the MD trajectory. The spread of the points shows the extent to which each internal molecular geometry distorts across the whole trajectory. Larger distortions result in a broader mist.

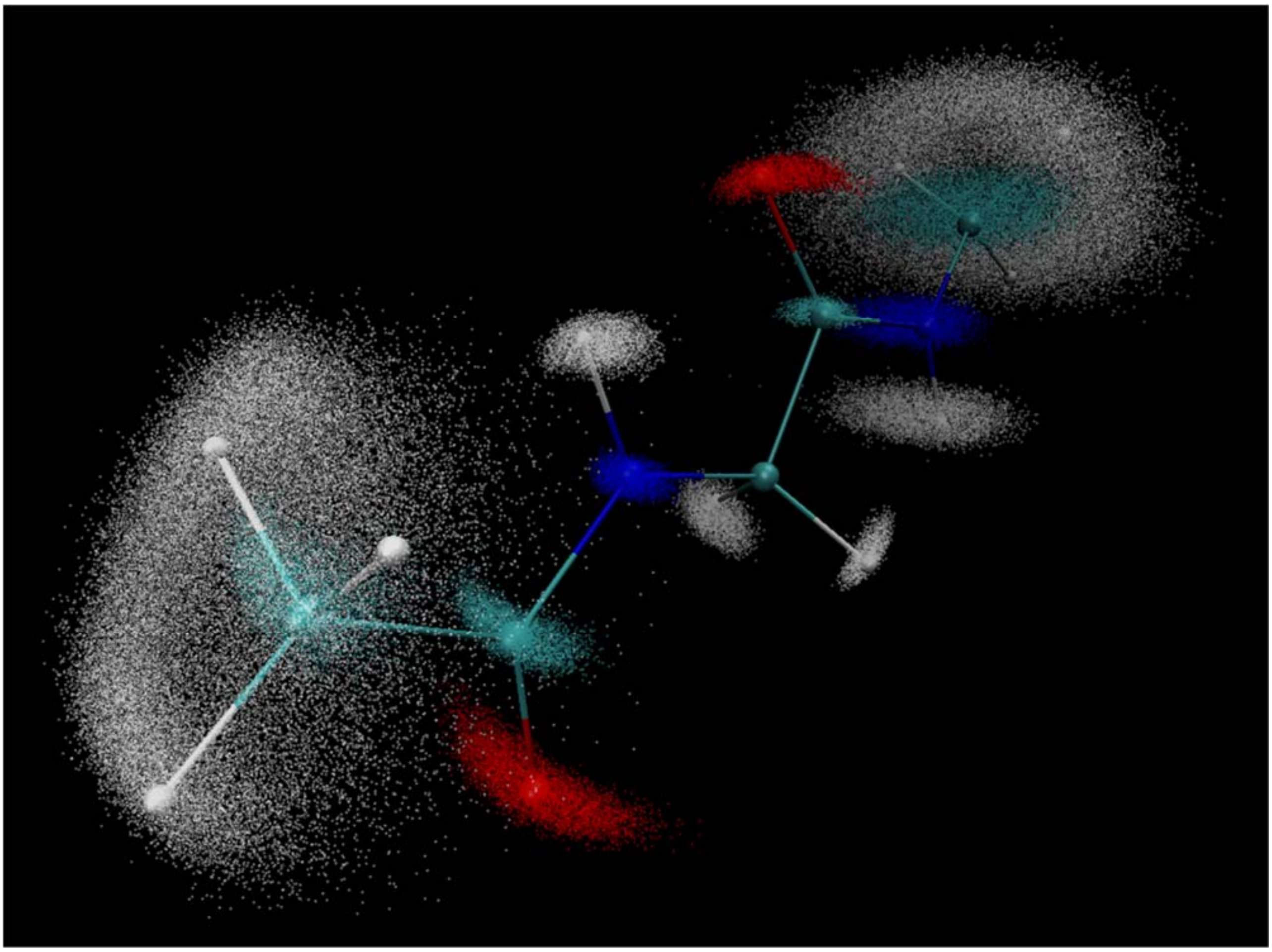

Figure S3.1 Peptide-capped glycine: AMBER 300 K mist plot.

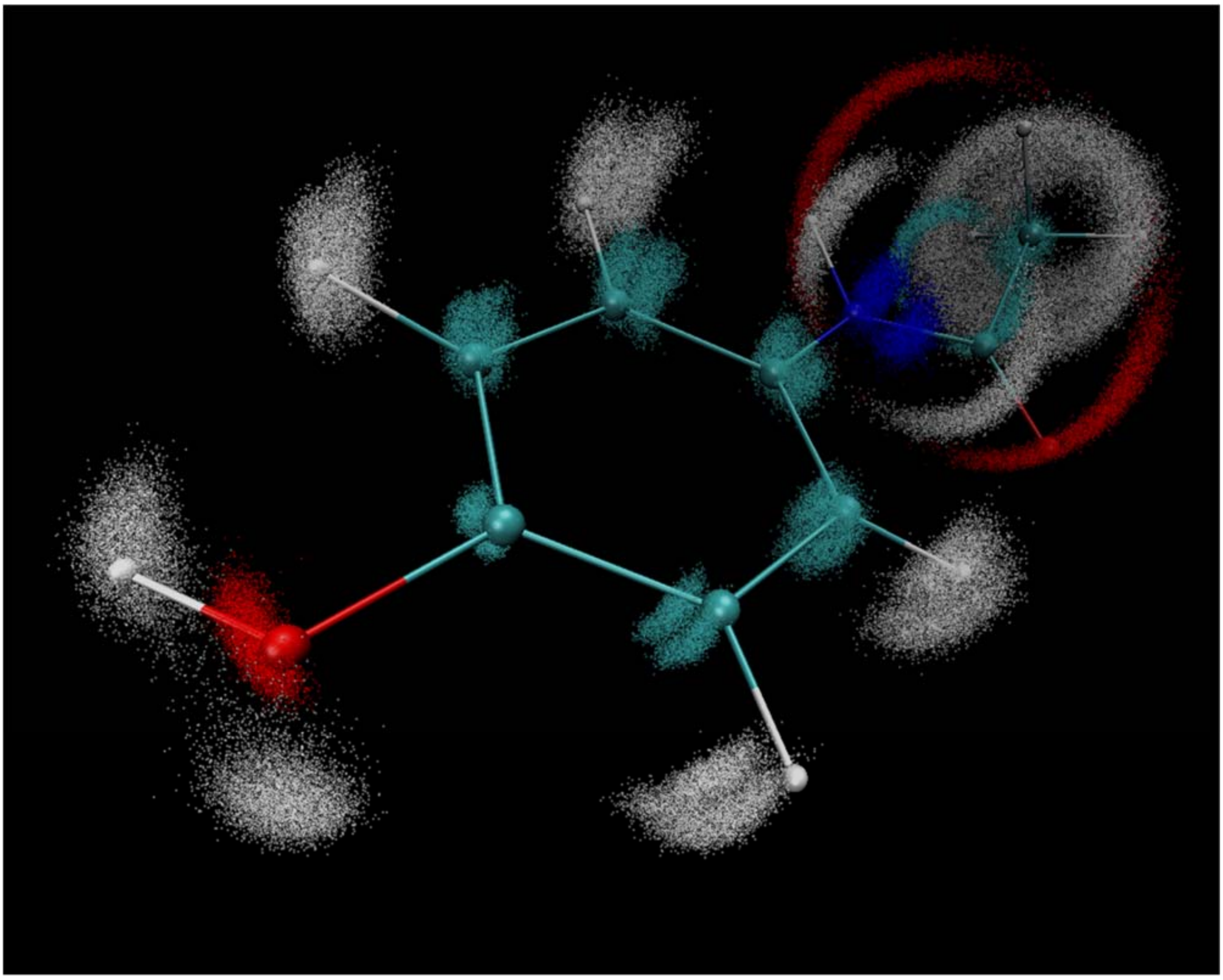

Figure S3.2 Paracetamol: AMBER 300 K mist plot.

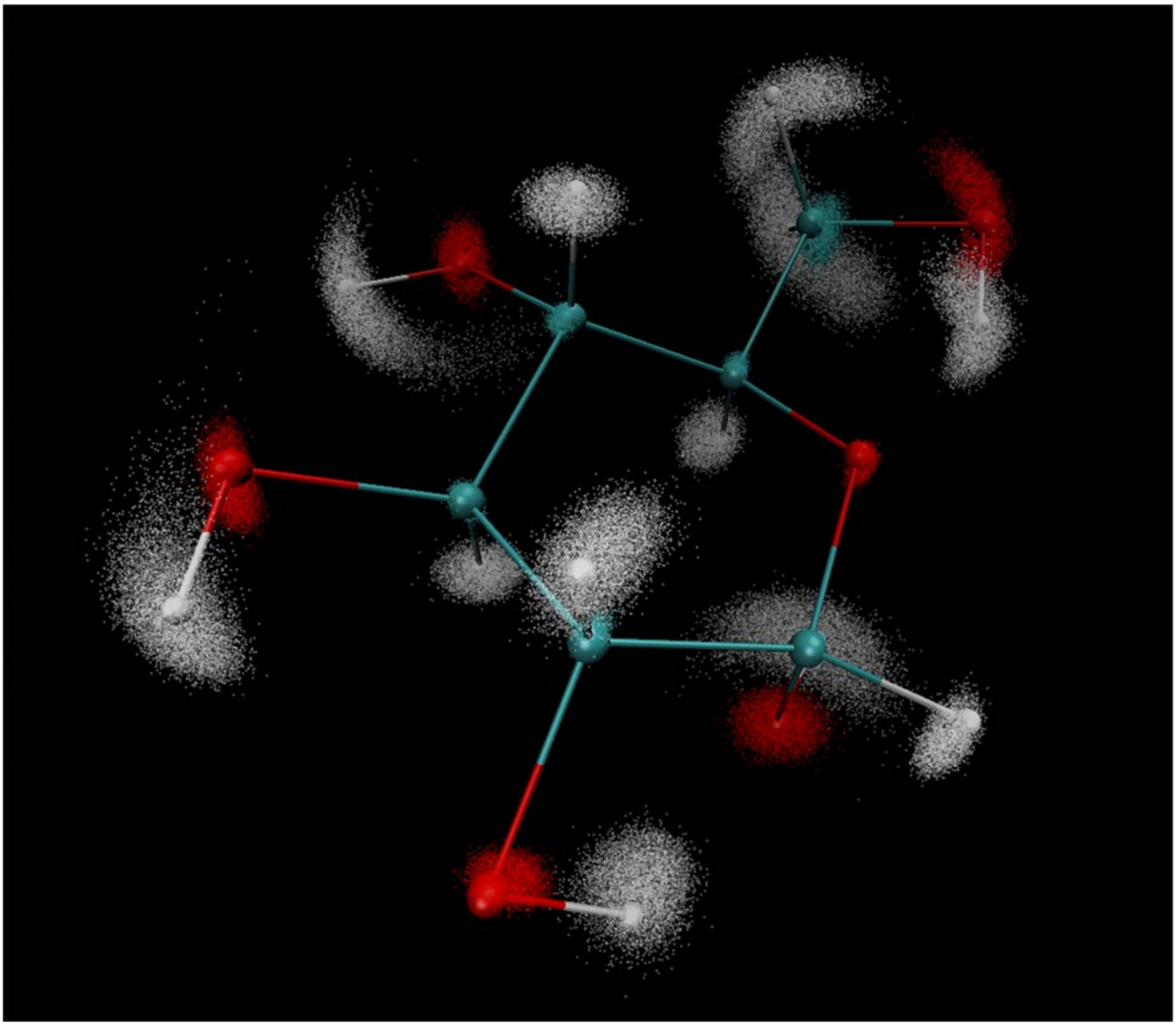

Figure S3.3 Glucose: AMBER 300 K mist plot.

4 S-Curves

S-curves visualise the absolute predictive accuracy of atomic properties. The S-surves display the full behaviour of a given model's performance over the whole validation set. Dipole moments and higher rank moments are tensor quantities, which makes their error analysis difficult to interpret. The multipole moments come together in a single formula expressing the electrostatic interaction.

In the interest of conciseness and clarity, the main text only shows simplified S-curves of the IQA energies of glycine and paracetamol (Figures 1 and 2), repeated here in full for sake of completeness, and the charge (Figure 4). The IQA S-curves for the remaining systems (aspirin, alanine, glucose and ibuprofen) can be found in Figures S4.3-S4.6 and Figures S4.7 and S4.8 give the remaining S-curves of all multipole moments (grouped by components), displayed as total prediction errors (using equation 23 from the main text).

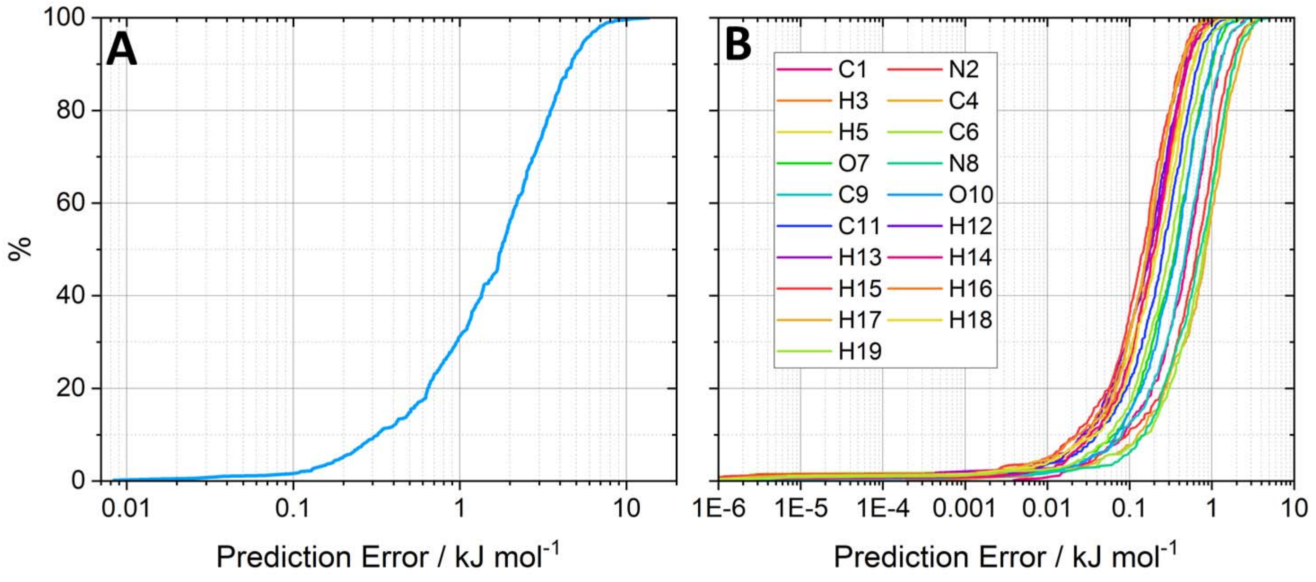

Figure S4.1 S-curves showing (a) the total IQA prediction error, and (b) individual atom IQA prediction errors for a glycine AMBER 300 K model.

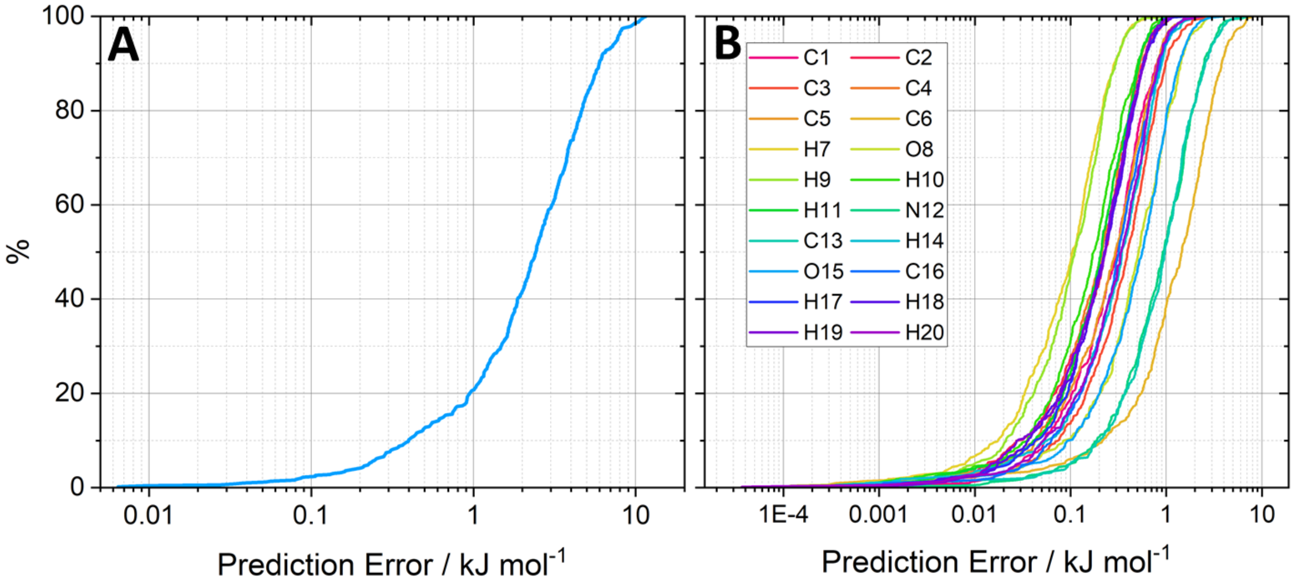

Figure S4.2. S-curves showing the (a) total IQA prediction error, and (b) individual atom IQA prediction errors for a paracetamol AMBER 300 K model.

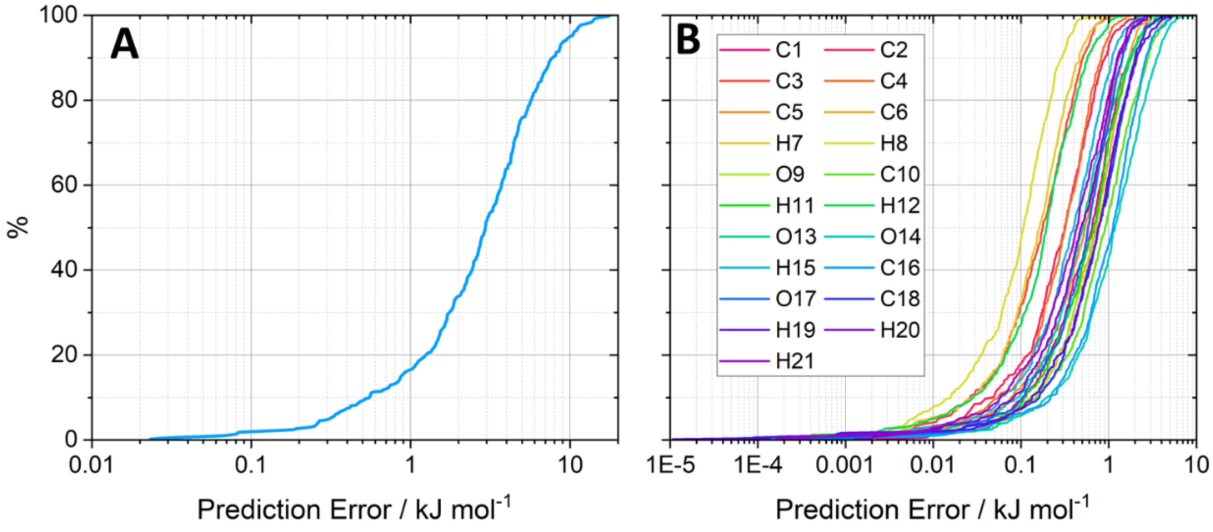

Figure S4.2. S-curves showing the (a) total IQA prediction error, and (b) individual atom IQA prediction errors for an aspirin AMBER 300 K model.

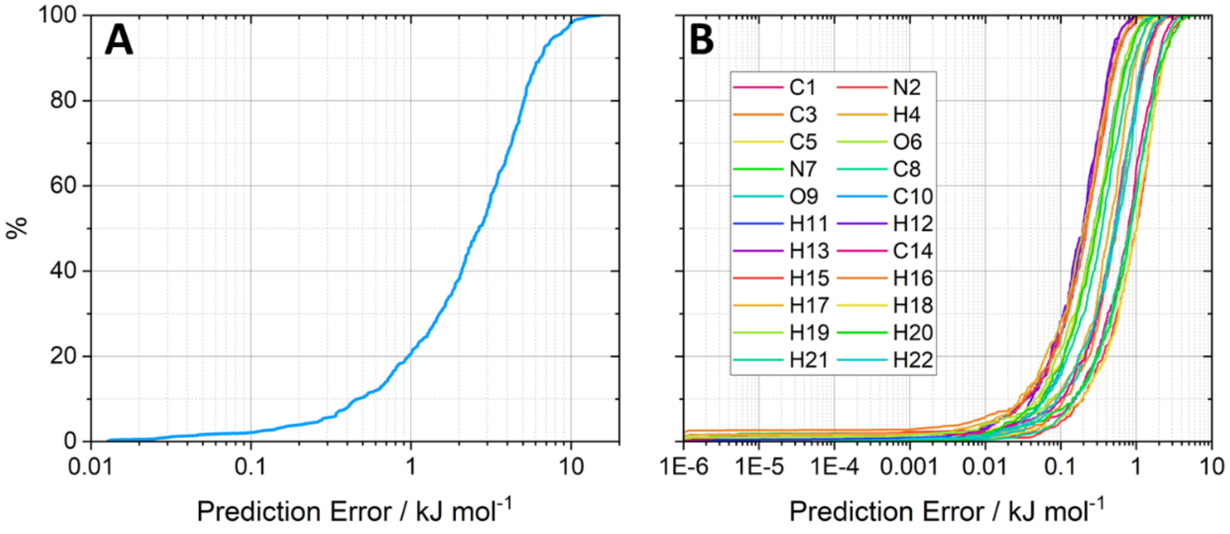

Figure S4.4 S-curves showing the (a) total IQA prediction error, and (b) individual atom IQA prediction errors for an alanine AMBER 300 K model.

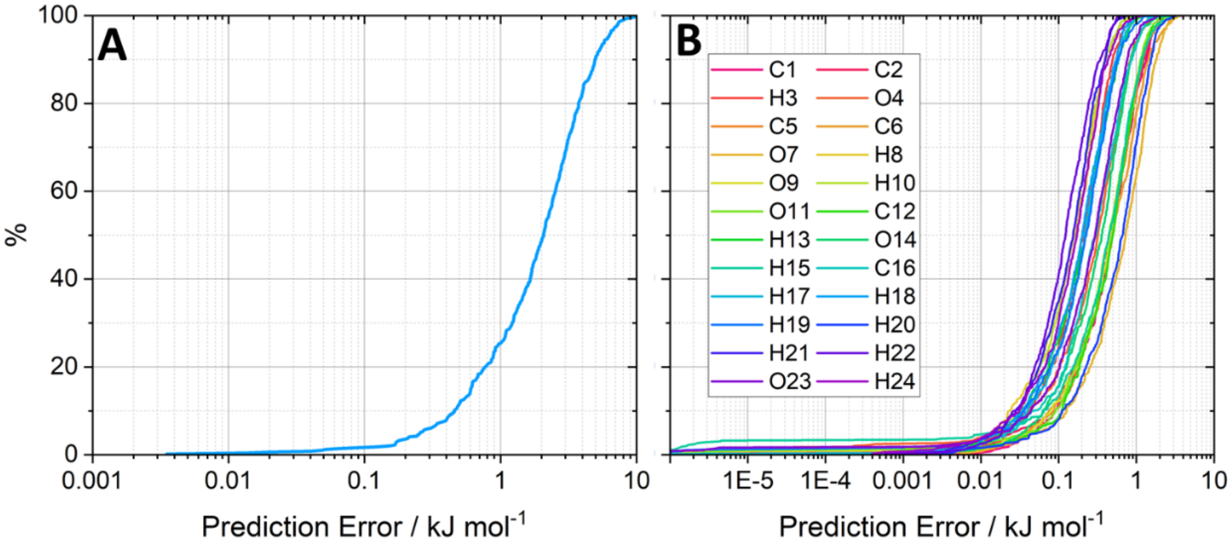

Figure S4.5 S-curves showing the (a) total IQA prediction error, and (b) individual atom IQA prediction errors for a glucose AMBER 300 K model.

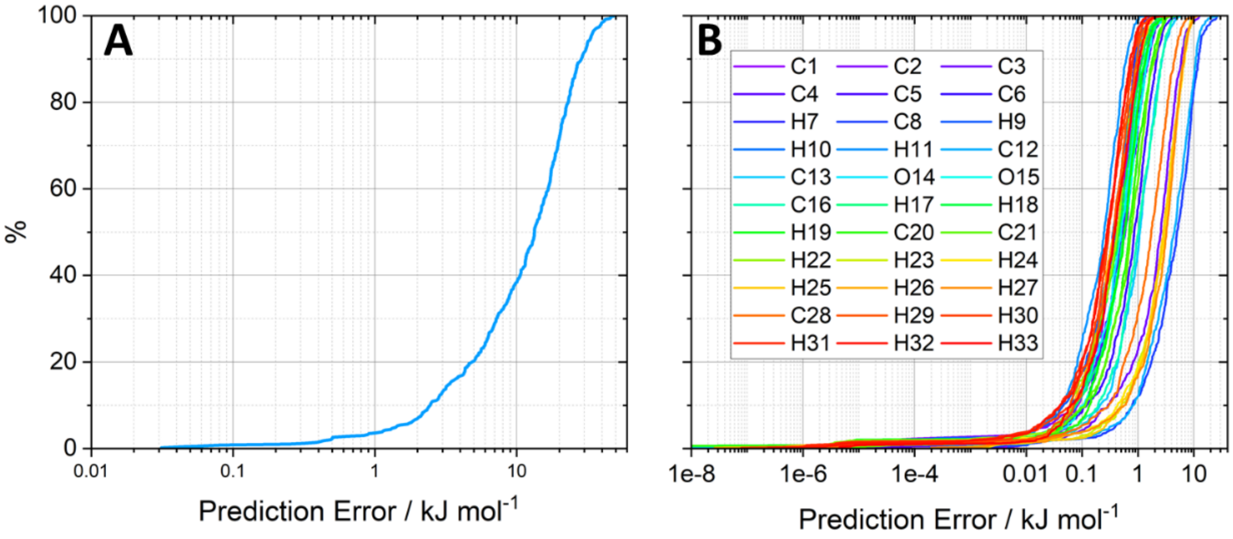

Figure S4.6 S-curves showing the (a) total IQA prediction error and (b) individual atom IQA prediction errors for an ibuprofen AMBER 300 K model.

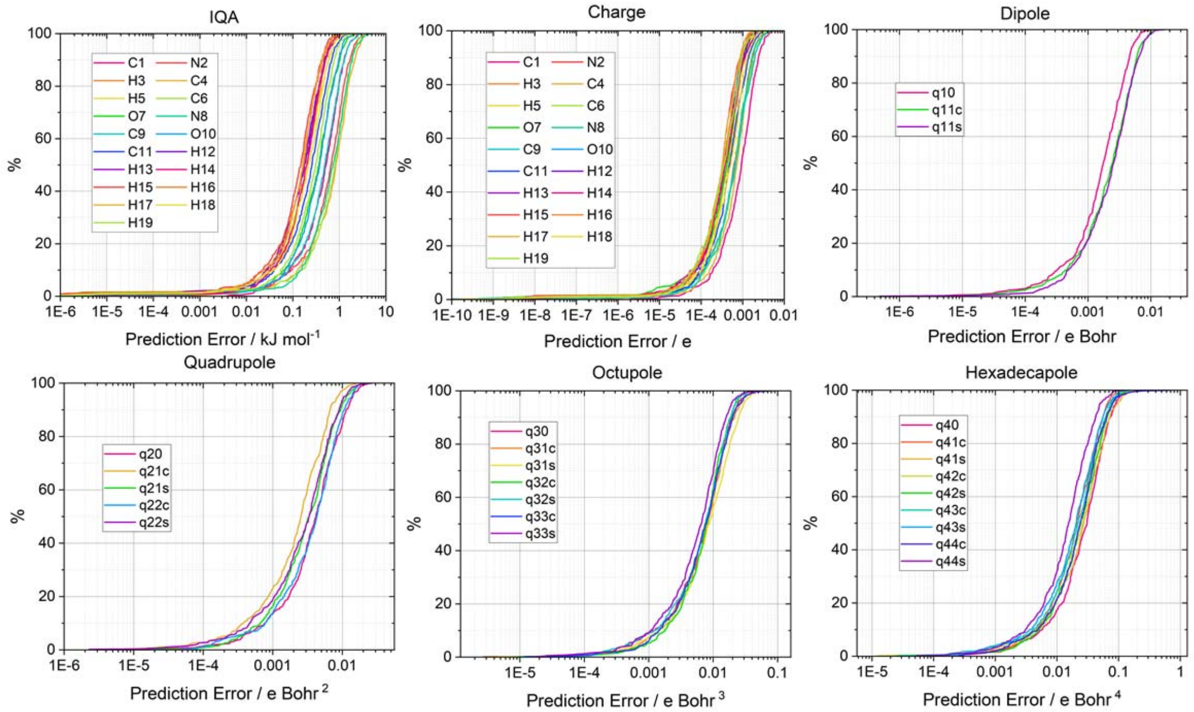

Figure S4.7 IQA energy and multipole moment S-curves for double-capped glycine. The prediction errors of the IQA and energy are shown for each atom while the prediction errors for all multipole moments (from dipole to hexadecapole) are shown as total prediction errors.

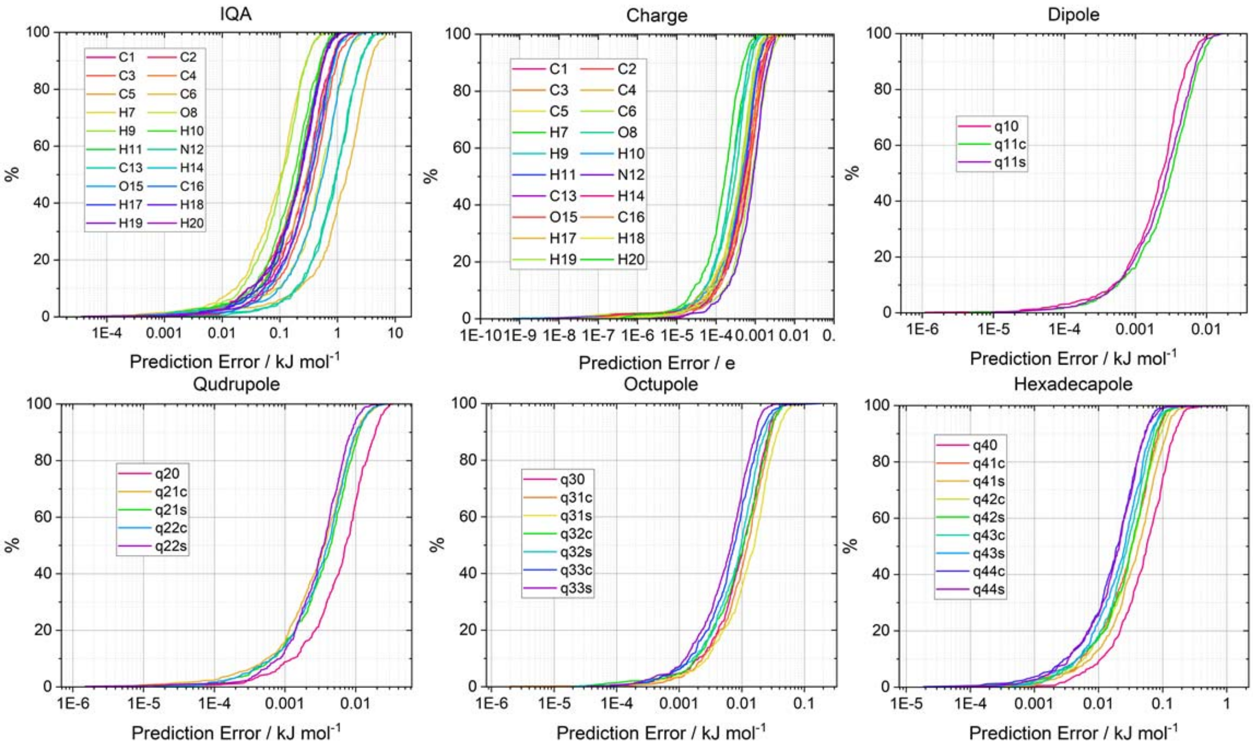

Figure S4.8 IQA energy and multipole moment S-curves for paracetamol. The prediction errors of the IQA and energy are shown for each atom while the prediction errors for all multipole moments (from dipole to hexadecapole) are shown as total prediction errors.

5 Atomic Local Frame Features

The atomic local frame (ALF) defines the axis system relative to an atom, thereby allowing the calculation of translationally and rotationally invariant features. The ALF features consist of 3 features associated with the atoms defining the ALF: distance to x-axis atom, distance to xy-plane atom and χ angle subtending the x-axis atom, the origin atom and the xy-plane atom. The remaining features are spherical polar coordinates for each non-ALF atom with respect to the ALF.

$$A_x = \sqrt{(\boldsymbol{A}_x - \boldsymbol{A}_o)^2} \tag{S1}$$

$$A_{xy} = \sqrt{\left(\boldsymbol{A}_{xy} - \boldsymbol{A}_o\right)^2} \tag{S2}$$

$$\chi^A = \cos^{-1}\left(\frac{\boldsymbol{A}_x.\boldsymbol{A}_{xy}}{A_x A_{xy}}\right) \tag{S3}$$

$$A_n = \sqrt{(\boldsymbol{A}_n - \boldsymbol{A}_o)^2} \tag{S4}$$

$$\theta^{An} = \cos^{-1}\left(\frac{\zeta_3^{\boldsymbol{A}_n}}{A_n}\right) \tag{S5}$$

$$\phi^{An} = \tan^{-1}\left(\frac{\zeta_2^{\boldsymbol{A}_n}}{\zeta_1^{\boldsymbol{A}_n}}\right) \tag{S6}$$

$$\text{where } n = (4, \dots, N)$$

where $\boldsymbol{\zeta}$ denotes the vector of the atom, n, rotated onto the ALF using rotation matrix $\boldsymbol{C}$,

$$\boldsymbol{\zeta} = \boldsymbol{C}(\boldsymbol{A}_n - \boldsymbol{A}_o) \tag{S7}$$

and the rotation matrix consists of the following three unit row vectors,

$$\boldsymbol{C}_1 = \frac{(\boldsymbol{A}_x - \boldsymbol{A}_o)}{R^{A_x}} \tag{S8}$$

$$\boldsymbol{C}_2 = \frac{\boldsymbol{y}}{\sqrt{\boldsymbol{y} \cdot \boldsymbol{y}}} \tag{S9}$$

$$\boldsymbol{C}_3 = \boldsymbol{C}_1 \times \boldsymbol{C}_2 \tag{S10}$$

where $\boldsymbol{y}$ is the vector respresenting the y-axis of the ALF

6 Particle Swarm Optimisation (PSO)

The stopping criterion used to termimate the PSO algorithm is the *relative change* stopping criterion. The relative change algorithm computes the relative difference, which is defined as the difference between the current iteration's optimum value and the previous iteration's optimum value, divided by the previous iteration's optimum value. Using the relative difference allows for the assignment of a fixed tolerance value without the necessity to regard the scale of the absolute optimum value, which for a GPR model is affected by the number of training points. If the relative change is beneath a tolerance value (δ_{tol}) for the predefined number of stall iterations (n_{stall}), then the PSO algorithm is deemed converged to an optimum value, and the optimisation is terminated.

$$\text{rel. diff.}\,(t) = \left| \frac{f\left(\boldsymbol{p}_{gb}(t)\right) - f\left(\boldsymbol{p}_{gb}(t-1)\right)}{f\left(\boldsymbol{p}_{gb}(t-1)\right)} \right| \tag{S11}$$

where f is the function being optimised and $\boldsymbol{p}_{gb}(t)$ is the global best position for iteration t.

Table S4. Parameters used for the PSO in FEREBUS.

**Parameter**	**Description**	**Value**
$N_{particle}$	Number of particles in the swarm	50
ω	Inertia-weight	0.729
c_1	Cognitive learning rate	1.494
c_2	Social learning rate	1.494
δ_{tol}	Relative difference convergence tolerance	1×10^{-8}
n_{stall}	Number of stall iterations before termination	50
n_{iter}	Maximum number of iterations	1,000
ε	Nugget (noise) parameter	1×10^{-8}
θ_{min}	Minimum value for each dimension of each particle	0.0
θ_{max}	Maximum value for each dimension of each particle	3.0

7 ICHOR

The program ICHOR is responsible for the generation of GPR models, active learning and analysis of the GPR models. ICHOR is an automated pipeline utility (to be more precise a library, as version 3.1) designed to generate QTAIM training data by interfacing with external programs and then using these data to generate GPR training sets for FEREBUS to use. Below is a schematic of a standard per-system active learning run.

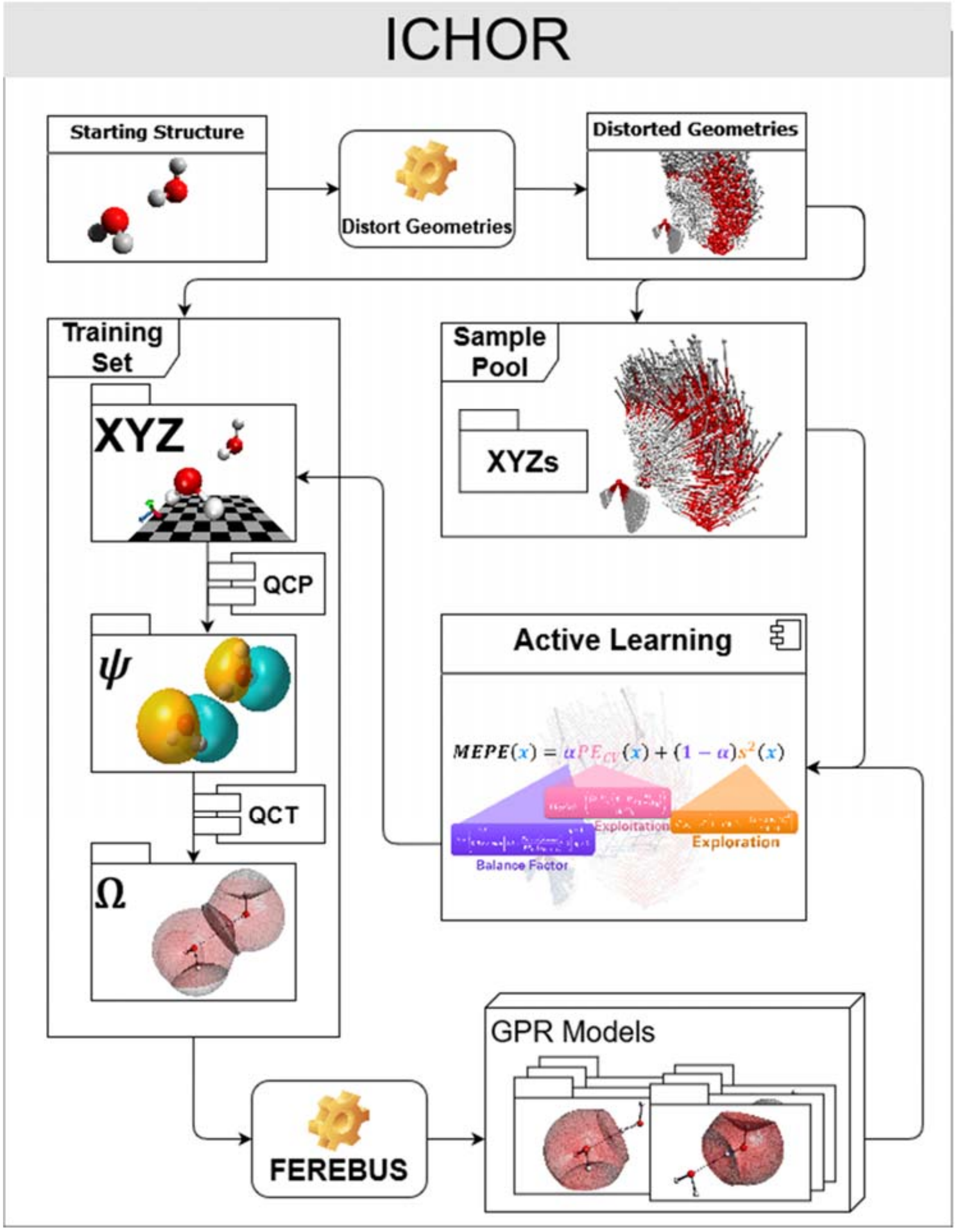

Figure S7.1 A schematic example of a per-system active learning run for a water dimer using ICHOR.
